# Supplementary material for: Exploring people’s thoughts about the causes of ethnic stereotypes
Source: PLoS One. 2021 Jan 19;16(1):e0245517. doi: 10.1371/journal.pone.0245517 (PMC7815097; doi:10.1371/journal.pone.0245517)
Supplement: S1 File — (DOCX) [file pone.0245517.s001.docx]

**SOM**

**Exploring people’s thoughts about the causes of ethnic stereotypes**

Study 1

**Articles participants read and attention check questions**

Note that correct response option is bolded; questions 6 and 7 are about participants’ opinion and were not analyzed.

**Genetic Dissimilarity condition:**


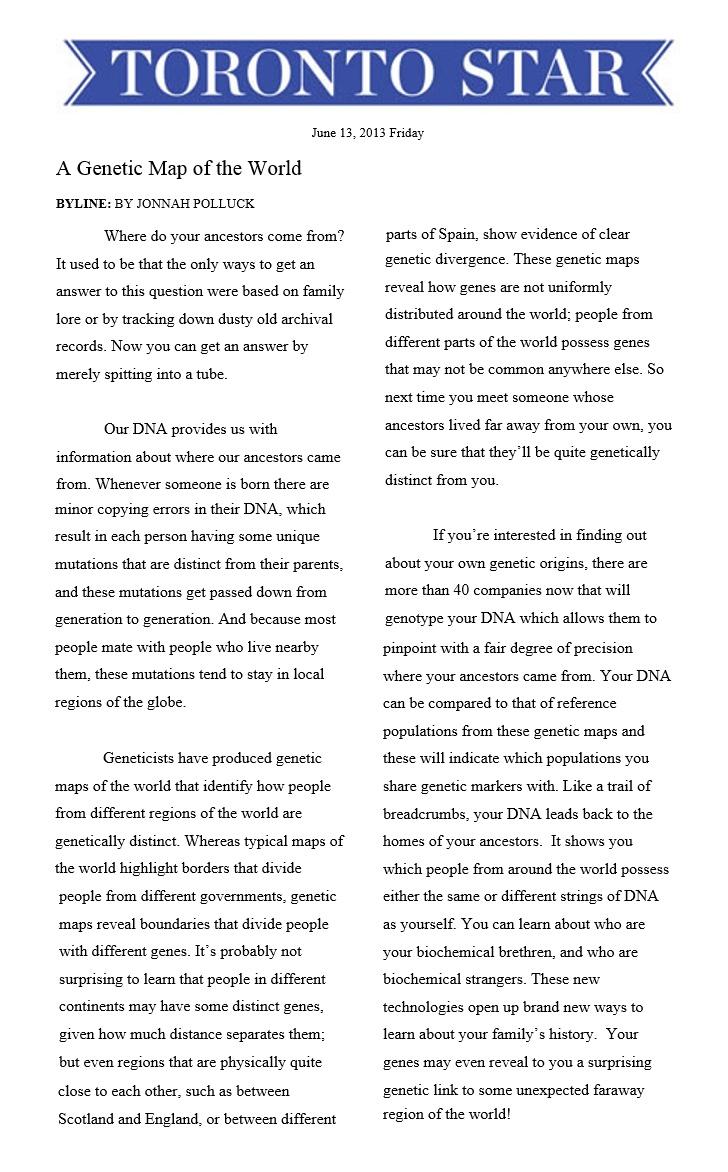


1. What is the method used to collect DNA in this essay?

**1.** **Spitting into a tube**

2. Cheek swab

3. Hair follicle sample

4. Blood sample

2. Which types of boundaries do genetic maps divide?

1. People with mental disorders

2. People with different cultures

**3.** **People with different genes**

4. People with genetic abnormalities

3. The further away someone's ancestors live from you:

**1.** **The more genetically different they are**

2. The more you'll look like them

3. The more genetically similar they are

4. The more you'll look like them

4. How many companies out there offer a service to genotype DNA?

1. 10

**2. 40**

3. 60

4. 5

5. Which branch of science involves the process of genotyping DNA?

1. Meteorology

2. Neurology

3. Psychoanalysis

**4. Biochemistry**

6. If I had the choice of getting my DNA genotyped:

1. I would get it done

2. I would not get it done

7. I think human beings are, for the most part:

1. Genetically similar to one another

2. Genetically different from one another

3. Connected by a force deeper than genetics

4. Naïve to how their DNA connects them to those around them

**Genetic Similarity condition:**


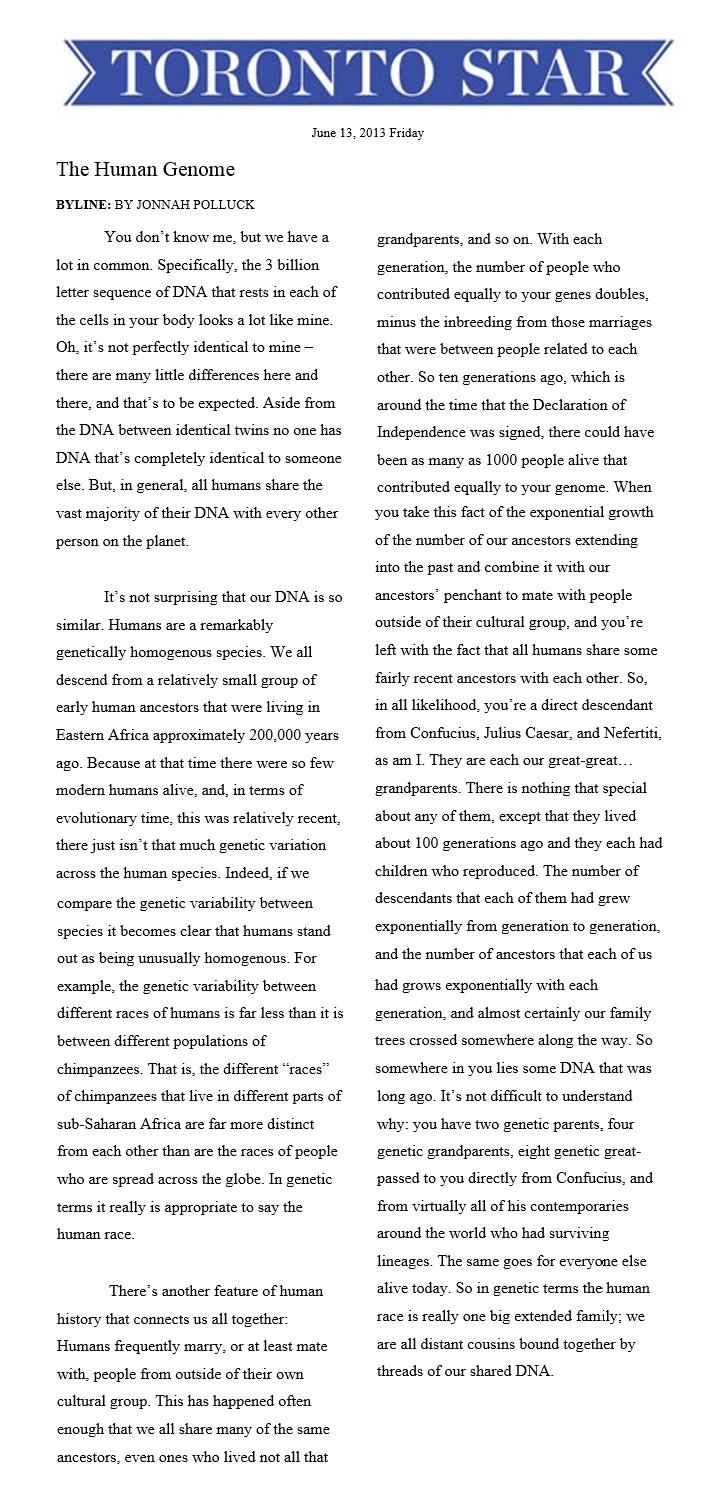


1. How long is the letter sequence of DNA that rests in human cells?

**1.** **3 billion sequences**

2. 10 billion sequences

3. 1 million sequences

4. 10,000 sequences

2. How long ago can our ancestors from Eastern Africa be traced back?

1. 10,000 years

2. 50,000 years

**3.** **200,000 years**

4. 1,000,000 years

3. Compared to chimpanzees, the genetic variation of humans is:

**1.** **Unusually similar to one another**

2. Extremely different from one another

3. Same degree of similarity as chimpanzees

4. Genetic variation of humans is not compared to chimpanzees

4. Humans are said to be connected, for the most part, due to:

1. The invention of boats and airplanes

**2.** **Reproducing with people from outside their own cultural group**

3. The internet

4. Eating food with similar chemical and nutritional components

5. Ten generations ago, how many people were alive that contributed equally to your genome?

1. 2

2. 10

3. 400

**4. 1,000**

6. I would be happiest if I found out I was related to:

1. Nefertiti

2. Elvis Presley

3. Sigmund Freud

4. Julius Caesar

7. I think human beings are, for the most part:

1. Genetically similar to one another

2. Genetically different from one another

3. Connected by a force deeper than genetics

4. Naïve to how their DNA connects them to those around them

**Control condition:**


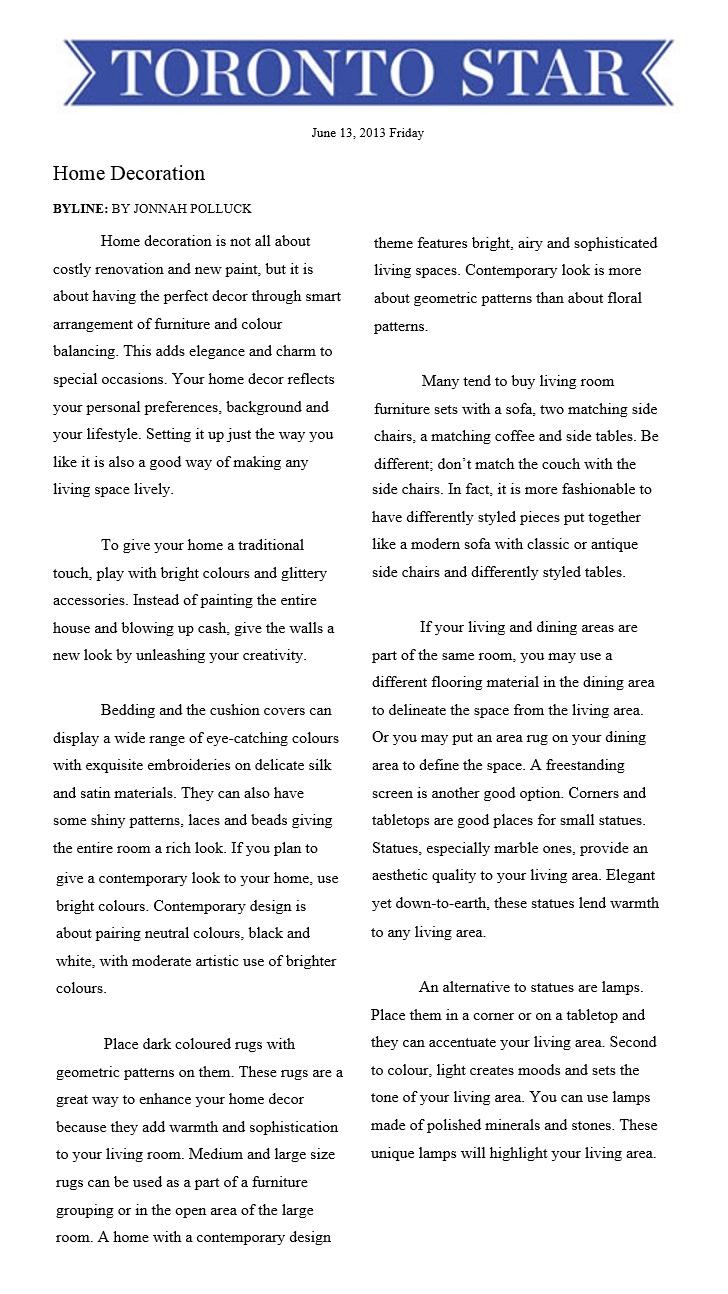


1. When using a contemporary design, what should you pair neutral colors with?

**1.** **Bright colors**

2. Dull colors

3. Pastel colors

4. Neon colors

2. What is not commonplace in a Contemporary room?

1. Sequence patterns

2. Striped patterns

**3.** **Floral patterns**

4. Geometric patterns

3. Which rugs look best with geometric patterns?

**1.** **Dark colored rugs**

2. Light colored rugs

3. Long threaded rugs

4. Short threaded rugs

4. What type of statues are said to provide an 'aesthetic quality' to your living area?

1. Glass statues

**2.** **Marble statues**

3. Silver statues

4. Clay statues

5. Which material lamps are said to be 'unique' and 'highlighting'?

**1. Polished minerals**

**2.** **Stones**

3. Tin

4. Both polished minerals and tin

* Note that both answers 1 and 2 were coded as correct

6. I would describe my own style of home décor as:

1. Contemporary

2. Modern

3. Rustic

4. Classic

7. When choosing the wall colors of a room, I prefer:

1. Light, neutral colors

2. Cool, calm colors

3. Eccentric, flashy colors

4. Dark, warm colors

**Table S1. Stereotypes Study 1, Mean accuracy, genetic, and environmental causes for stereotypes broken down by stereotype**

|  | Stereotype | Mean Accuracy (SD) | Mean genetic attribution (SD) | Mean environmental attribution (SD) |
| --- | --- | --- | --- | --- |
| 1. | The Dutch are, on average, taller than people from most other countries. | 4.07 (1.44) | 75.85% (16.49%) | 36.92% (26.38%) |
| 2. | Japanese have longer lifespans than people from most other countries. | 5.02 (1.29) | 61.66% (22.69%) | 64.39% (24.63%) |
| 3. | East Asians are better at math than people from most other countries. | 4.46 (1.42) | 45.66% (27.48%) | 69.03% (25.29%) |
| 4. | Native Americans are better hunters than people from most other industrialized countries. | 4.15 (1.45) | 42.06% (26.10%) | 76.94% (18.72%) |
| 5. | Indians enjoy eating spicier foods than people from most other countries. | 4.69 (1.33) | 38.38% (27.18%) | 75.41% (21.22%) |
| 6. | On average, French have a more refined palate than people from most other countries, and they prefer the taste of high quality cuisine. | 3.68 (1.49) | 35.12% (27.85%) | 74.35% (22.48%) |
| 7. | Africans have a better sense of rhythm than people from most other countries. | 3.94 (1.62) | 48.68% (28.26%) | 66.07% (25.20%) |
| 8. | On average, Germans are more efficient and rule-abiding than people from most other countries. | 3.66 (1.55) | 33.73% (27.50%) | 76.52% (22.52%) |
| 9. | Brazilians are better at soccer than people from most other countries. | 3.97 (1.50) | 40.68% (27.43%) | 72.38% (21.72%) |
| 10. | The Japanese are more interested in robots than are people from most other countries. | 3.84 (1.56) | 27.13% (27.11%) | 76.29% (24.36%) |

Study 2

**Table S2. Stereotypes Study 2, Mean accuracy, Mean Offense, genetic, and environmental causes for stereotypes broken down by stereotype**

|  | Stereotype | Mean Accuracy (SD) | Mean genetic attribution (SD) | Mean environmental attribution (SD) |
| --- | --- | --- | --- | --- |
| 1. | On average, French people have a more refined palate than people from most other countries, and they prefer the taste of high quality cuisine. | 3.78 (1.50) | 33.77% (26.85%) | 72.92% (23.34%) |
| 2. | Japanese have longer lifespans than people from most other countries. | 4.92 (1.46) | 57.73% (26.34%) | 68.66% (22.52%) |
| 3. | Africans have a better sense of rhythm than people from most other countries. | 3.92 (1.63) | 47.14% (29.55%) | 66.47% (25.59%) |
| 4. | Native Americans are better hunters than people from most other industrialized countries. | 3.96 (1.70) | 38.91% (29.06%) | 79.05% (20.40%) |
| 5. | Brazilians are better at soccer than people from most other countries. | 3.87 (1.64) | 35.43% (28.08%) | 75.20% (22.78%) |
| 6. | Swedish people are more attractive than people from most other countries. | 3.34 (1.67) | 81.22% (17.53%) | 33.92% (25.72%) |
| 7. | African Americans are better basketball players than people from most other countries. | 4.50 (1.67) | 64.75% (25.51%) | 56.95% (27.47%) |
| 8. | Italians are, on average, more emotionally expressive than people from most other countries. | 3.93 (1.56) | 40.43% (29.58%) | 71.39% (24.46%) |
| 9. | Americans are more patriotic than people from most other countries. | 3.75 (1.81) | 19.71% (25.57%) | 85.13% (20.71%) |
| 10. | The Japanese are more interested in robots than are people from most other countries. | 3.66 (1.64) | 24.82% (25.67%) | 79.07% (22.06%) |
| 11. | On average, Germans are more efficient than people from most other countries. | 3.44 (1.65) | 36.71% (27.96%) | 74.35% (22.20%) |
| 12. | On average, Canadians are more polite than people from most other countries. | 4.06 (1.73) | 25.74% (27.28%) | 81.61% (21.75%) |
| 13. | Russians are more militaristic than people from most other countries. | 3.71 (1.58) | 27.04% (26.58%) | 81.90% (19.75%) |
| 14. | Jews are funnier than people from most other countries. | 2.64 (1.51) | 35.39% (28.26%) | 72.28% (24.16%) |
| 15. | The Irish like to drink more than people from most other countries. | 4.15 (1.71) | 49.76% (30.01%) | 73.38% (23.14%) |
| 16. | On average, Asians are worse drivers than are people from most other countries. | 3.33 (1.82) | 39.14% (30.88%) | 70.12% (26.03%) |
| 17. | English people have, on average, worse teeth than people from most other industrialized countries. | 3.62 (1.69) | 59.23% (30.61%) | 64.17% (27.74%) |
| 18. | On average, Chinese are ruder than people from most other countries. | 3.16 (1.73) | 30.13% (28.52%) | 79.52% (22.21%) |
| 19. | Mexicans are, on average, lazier than people from most other countries. | 2.06 (1.42) | 44.50% (28.25%) | 67.51% (24.36%) |
| 20. | On average, African-Americans aren’t as intelligent as people from most other countries. | 2.21 (1.64) | 58.41% (29.55%) | 67.98% (27.40%) |
