# Supplementary material for: Exploring people’s thoughts about the causes of ethnic stereotypes
Source: PLoS One. 2021 Jan 19;16(1):e0245517. doi: 10.1371/journal.pone.0245517 (PMC7815097; doi:10.1371/journal.pone.0245517)
Supplement: Attachment — Submitted filename: PLOS ONE Article Review 11-2020.docx [file pone.0245517.s002.docx]

**Summary and General Impression:**

Across two studies, the researchers examined how ideas of genetic essentialism impact the perceived causes of ethnic stereotypes. In Study 1, participants read one of two articles which described the ability to estimate ancestry through genetics or the homogeneity of the human genome, or read a control article discussing an unrelated topic. They were then asked to describe the extent to which ethnic stereotypes were caused by genetic and environmental factors on a numerical scale. Among participants who believed ethnic stereotypes were accurate, those in the control condition and the ancestry via genetics condition viewed stereotypes as being more related to genetics and less related to environmental factors than those in the homogenous genome condition. Across conditions, however, participants rated environmental factors as the majority influence on ethnic stereotypes. Genetic essentialist beliefs were also associated with greater attribution of genetics to stereotypes.

In Study 2, the authors replicated Study 1 with some additions. They expanded their list of stereotypes, included a measure of knowledge about genetics, and measured attitudes towards the ethnic groups described in the list of stereotypes. The finding that those in the genetic ancestry condition were more likely to attribute stereotypes to genetic causes was replicated; however, those in the control condition were most similar to the homogenous genome condition, unlike Study 1.

Overall, the manuscript is well-written and well-organized. It provides an interesting extension to past research on genetic essentialism and racial attitudes by examining the influence of genetic information on perceptions of the origins of stereotypical behavior. Both studies use large sample sizes and the procedures for excluding participants are well-explained. The statistics used are appropriate and corrections for multiple comparisons and effect sizes are clearly present. The authors present interesting ideas on the real-world implications of this research given the popularity of ancestry-related genomic testing and the ability for increased knowledge about genes as a means of reducing essentialist attitudes.

**Suggestions for Improvement**

1. The general discussion discusses three major findings: that reading about genetic differences used to determine ancestry increases essentialist views of stereotypes, that people have essentialist views of genetics in general, and that overall genetic knowledge is low. In my read, the support for the first conclusion is strong. However, the scores for the first genetic essentialism scale are just above the midpoint in both studies. I would appreciate more explanation of how this finding is interpreted as generally more essentialist views.
2. Similarly, the authors state that overall genetic knowledge is low. This was measured only in Study 2, with a score of just over 6 / 9 correct. While this does not indicate a high level of knowledge about genes, I don’t know that I would characterize this as a low level of knowledge.
3. Overall, the discussion is quite short. I’d like to see some discussion of how the results of this research build upon and/or replicate previous work in this area.
4. Both studies include an analysis of the effects of social dominance orientation. However, it is not explained why this variable is included and social dominance orientation is not discussed in the introduction.
5. Information about participants’ racial/ethnic backgrounds is not provided. If this is available, it should be included in the participants section.
6. I would appreciate more information about the development of the stereotypes for both studies. How were these stereotypes selected? Was there any consideration of differential interpretations for stereotypes related to physical appearance compared to stereotypes about habits, personality, etc. It seems it would be easier to mentally link some of these stereotypes to genes compared to others.
7. Minor – There are numerous issues with APA format, particularly in the References section
